# A Systematic Review and Meta-Analysis on the Occurrence of Toxoplasmosis in Animals Slaughtered in Brazilian Abattoirs

**DOI:** 10.3390/ani12223102

**Published:** 2022-11-10

**Authors:** Gabriel Augusto Marques Rossi, Eduardo de Freitas Costa, Sarah Gabriël, Fábio Ribeiro Braga

**Affiliations:** 1 University Vila Velha (UVV), Department of Veterinary Medicine, Avenue Comissário José Dantas de Melo, Number 21, Vila Velha 29102-920, Brazil; 2 Department of Epidemiology, Bio-Informatics and Animal Models, Wageningen Bioveterinary Research, 8221 RA Lelystad, The Netherlands; 3 Department of Translational Physiology, Infectiology and Public Health, Faculty of Veterinary Medicine, Ghent University, 9820 Merelbeke, Belgium

**Keywords:** abattoir, foodborne, meat, parasite, prevalence, risk factors

## Abstract

**Simple Summary:**

Toxoplasmosis is a zoonosis primarily transmitted via the consumption of infected/contaminated meat/vegetables/fruits, resulting in several years of disability-adjusted life. In Brazil, a high prevalence of toxoplasmosis has been recorded, and it affects hundreds of people. Several studies have been performed in Brazil to determine the occurrence of toxoplasmosis in animals at different levels of the food chain. However, to fully understand the situation in the country, there is a need to compile the studies performed in abattoirs establishing *Toxoplasma gondii* prevalence, its risk factors, as well as the infectivity of seropositive animals. Thus, we performed a systematic review gathering data on the prevalence and risk factors for toxoplasmosis in animals slaughtered in Brazil and performed a meta-analysis of its prevalence for different species and regions. Based on the results, we also discussed the infectivity of seropositive animals, risk factors, and the preventive strategies to avoid this zoonosis.

**Abstract:**

Toxoplasmosis is mainly transmitted to human beings through the consumption of contaminated food, and several outbreaks caused by infected meat consumption have been reported in Brazil. We performed a systematic literature review on the prevalence and risk factors for toxoplasmosis in slaughtered animals and performed a meta-analysis of its prevalence for different species and regions. Furthermore, we also discussed the infectivity of seropositive animals, risk factors, and preventive strategies. In the meta-analysis, the overall prevalence estimates for poultry, ostrich, goats, swine, equids, sheep, and bovines were 42.4, 40.4, 23.0, 19.9, 19.1, 17.3, and 16.2%, respectively. Regarding the Brazilian regions, the highest prevalence values were detected for bovines and equids in the South (32.3 and 34.4%, respectively) and swine, goats, sheep, and poultry in the Northeast (29.3, 23.0, 22.9, and 69.8%%, respectively). High proportions of *Toxoplasma gondii* viability in bioassay conducted on seropositive animals were seen for sheep (34/40 = 85%) and swine (12/15 = 80%). *Toxoplasma gondii* infections are widespread on Brazilian farms, and the summarized data allow the establishment of high-priority areas and/or species for the adoption of preventive strategies to control this parasite at different levels of the food chain.

## 1. Introduction

*Toxoplasma gondii* is a protozoan parasite that infects most warm-blooded animals and humans. Its lifecycle is complex and involves members of the family *Felidae* as definitive hosts and mammals and avian as intermediate hosts. Felids shed numerous sporulated oocysts in their feces for approximately one to three weeks, thereby contaminating the environment. Later, these oocysts become infectious and can cause infection when ingested by intermediate hosts (warm-blooded animals and humans). In these hosts, oocysts turn into tachyzoites and, subsequently, into bradyzoites in neural and muscle tissues [1]. Toxoplasmosis is congenitally transmitted, but the transmission to humans (and other carnivores) as a foodborne disease is noteworthy and can occur in two distinct ways: via the ingestion of infective oocysts in contaminated water and food (mainly vegetables) or via the consumption of raw or undercooked meat containing cysts. This last route has been considered the most important [2].

Toxoplasmosis is a worldwide zoonosis, and the global annual incidence of the congenital disease has been estimated at 190,100 cases (1.5 per 1000 live births) and is equivalent to a burden of 1.20 million disability-adjusted life years [3]. In the United States, the annual burden of toxoplasmosis is around 10,964 quality-adjusted life years, and the cost of illness is about USD 2.9 billion [4]. Although outbreaks of clinical toxoplasmosis occur worldwide, they mainly occur in Brazil, including large outbreaks related to environmental contamination, poor hygiene, and poor socioeconomic conditions [5]. The consumption of contaminated meat represents the source of some of these outbreaks [5]. Additionally, atypical strains with a high virulence potential occur in this country [6].

The presence of *T. gondii* in meat sold in Brazil for human consumption has been frequently reported [7,8]. Indeed, several studies have been conducted into the occurrence of toxoplasma in animals slaughtered for human consumption in different regions in Brazil, a key meat producer and exporter country [9,10,11,12]. To enhance the current understanding of the local situation across the different regions in Brazil, a good overview using meta-analyses of all existing data is required. Furthermore, compiling the risk factors and the infectivity of seropositive animals using bioassay or PCR can help to understand its transmission.

Therefore, the aim of this study was (i) to compile data regarding the prevalence and risk factors for toxoplasmosis in animals sampled in Brazilian abattoirs via a systematic literature review; (ii) to subsequently perform a meta-analysis to estimate the toxoplasmosis prevalence for different animal species and regions; (iii) to discuss the seroprevalence results in relation to the infectivity of seropositive animals based on bioassay and/or PCR; and (iv) discuss useful strategies for controlling this parasite.

## 2. Methods

### 2.1. Study Area

**Figure 1:**
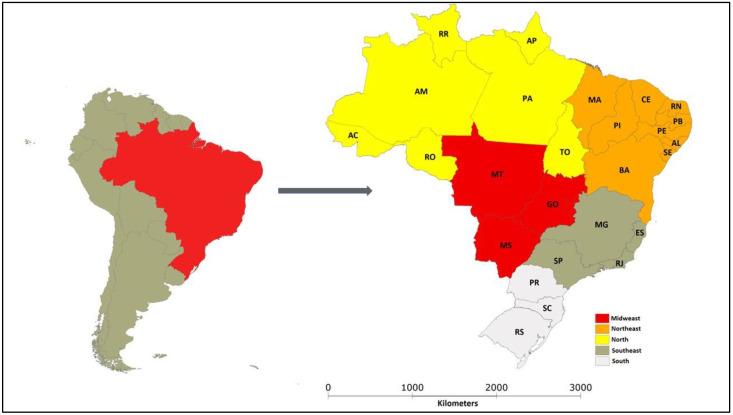
Maps showing the different regions and states in Brazil. Brazil is divided into the following states: Acre (AC), Alagoas (AL), Amapá (AP), Amazonas (AM), Bahia (BA), Cerá (CE), Espírito Santo (ES), Goiás (GO), Maranhão (MA), Mato Grosso (MT), Mato Grosso do Sul (MS), Minas Gerais (MG), Pará (PA), Paraíba (PB), Paraná (PR), Pernambuco (PE), Piauí (PI), Rio de Janeiro (RJ), Rio Grande do Norte (RN), Rio Grande do Sul (RS), Rondônia (RO), Roraima (RR), Santa Catarina (SC), São Paulo (SP), Sergipe (SE) and Tocantins (TO), which are grouped into five Brazilian regions (Midwest, Northeast, North, Southeast, and South).

Brazil is located in South America and has a surface area of 8 million km^2^, organized into 27 federative units: 26 states and 1 Federal District (DF) (located in Goiás State), organized in 5 regions covering 5570 municipalities (Figure 1).

The maps were created in Terraview^®^ Software (INPE, São José dos Campos, Brazil, v.4.2.0) (www.dpi.inpe.br/terraview, accessed on 12 October 2022)

### 2.2. Search Strategy

The authors performed a non-registered systematic review independently and followed the Preferred Reporting Items for Systematic Reviews and Meta-Analyses (PRISMA) guidelines (Table S1) [13]. A review of the published literature was conducted to obtain data about the prevalence and risk factors associated with toxoplasmosis in animals slaughtered for human consumption in Brazilian abattoirs. The keywords (“Toxoplasm*” AND “Brazil” AND “slaughter*”) were systematically searched in the following seven databases: *Scopus, ScienceDirect, SciELO, Redalyc, PubMed, Lilacs, and Cabdirect*. Additionally, we also used (“Toxoplasma” AND “Brasil” AND “Frigorífico”) (Portuguese) in *Redalyc*. All articles were last accessed on 13 August 2022.

**Figure 2:**
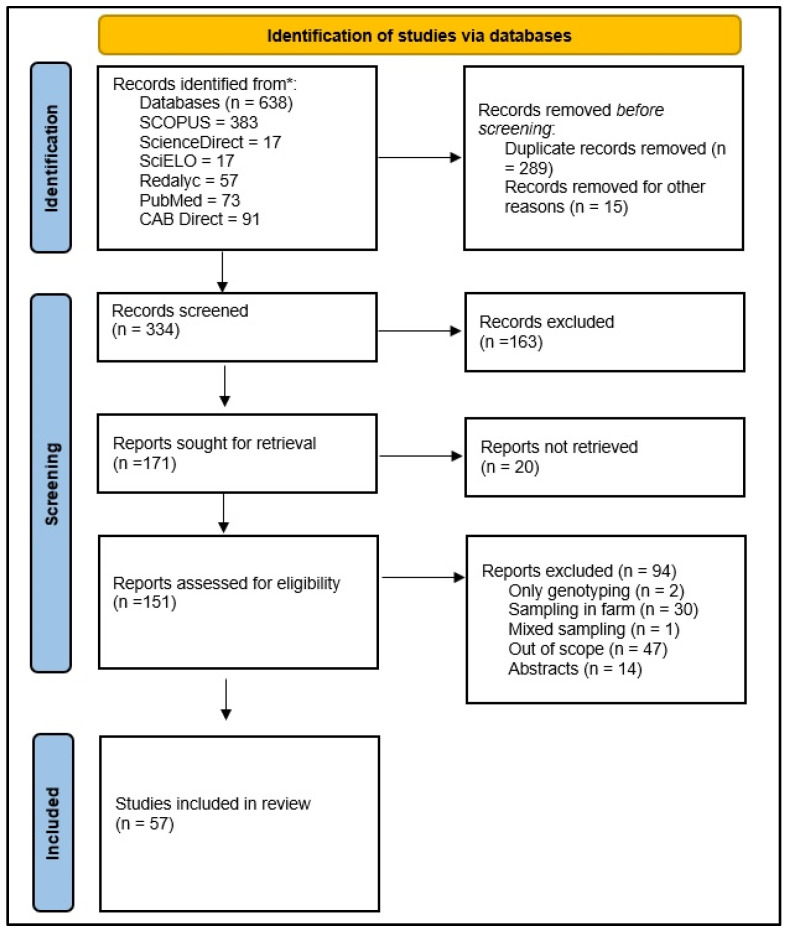
PRISMA flowchart diagram of the record selection process.

Subsequently, the compilation was performed by two authors, the duplicate records were removed, and the relevance of the results was analyzed (firstly scanning the title and abstract, and if applicable the full text). Manuscripts out of scope were removed in this phase. The following inclusion criteria were used to select articles: (i) studies performed in Brazilian abattoirs; (ii) published in peer review journals; (iii) full text available online in Portuguese or English; and (iv) containing information on toxoplasmosis prevalence or risk factors for animals (to be) slaughtered in Brazilian slaughterhouses. The articles considered non-eligible were reviews, manuscripts with no access to the full text, studies not performed in Brazil, and others lacking specifications on whether the study was performed on farms or in abattoirs (Figure 2).

### 2.3. Data Analysis

Quantitative data were stored in a predefined spreadsheet document, including the species, state, region, period of the study, number of sampled animals, number of infected and uninfected animals, prevalence, the sample used for diagnosis (blood or tissues), method of detection and risk factors (when available), authors and year of publication. When manuscripts used a serological method for diagnosis and additionally performed bioassay or PCR in tissues from seropositive animals, we also extracted data on the type of tissue, the number of positive and negative samples after additional testing, and proportion of positives (%, proportion of the number of seropositive animals testing positive on the bioassay/PCR and the number of seropositive animals).

Subsequently, two authors checked and compared the extracted data and compiled the final dataset of extracted data (Table S2). If divergences were detected, the authors decided together on the inclusion of these manuscripts and the extracted data.

For the meta-analyses, the location (region) of the abattoir was considered as the origin of animals when the exact origin was not provided, as in Brazil, most animals are slaughtered locally. Additionally, when animals came from states located in different regions, and there were no separate data for each state/region, these studies were not included in the meta-analysis of prevalence by regions/species. When any data were missing (i.e., the number of uninfected animals), we calculated them using the available data in the manuscript.

### 2.4. Meta-Analyses

A random-effects meta-analysis was conducted to assess the prevalence of toxoplasmosis in each animal species using geographical regions as a sub-group analysis. The data were logit-transformed, using an increment of 0.5 when the number of positive samples was zero. The maximum likelihood estimator for the expected prevalence was weighted using the sum of the inverse of the within-study variance and the between-study variance using the DerSimonian–Laird method. For studies reporting results for multiple states, data from different states within the same region were combined by summing the number of positive cases and the total number of animals. All analyses were conducted in R (R Core Team) [14] using the function *metraprop* from library *meta* [15]. The scripts and the data used for all analyses are available at: https://github.com/eduardodefreitascosta/Toxoplasmose (accessed on 13 August 2022).

## 3. Results

A total of 57 peer-reviewed published articles studying the prevalence of toxoplasmosis in animals slaughtered in Brazilian abattoirs were found (Table S2) [9,10,11,12,16,17,18,19,20,21,22,23,24,25,26,27,28,29,30,31,32,33,34,35,36,37,38,39,40,41,42,43,44,45,46,47,48,49,50,51,52,53,54,55,56,57,58,59,60,61,62,63,64,65,66,67,68]. These manuscripts covered species such as pigs (n = 30 manuscripts) (Table S3), cattle (n = 11), goats (n = 6), sheep (n = 11) (Table S4), equids (n = 4), chickens (n = 2) and ostriches (n = 2) (Table S5). Almost all of the studies used indirect immunofluorescence (IFAT) for detecting positive animals; however, the modified agglutination test (MAT), indirect hemagglutination, ELISA, Western blot, and PCR were used in a few studies (Table S1).

According to manuscripts found in the systematic review, the highest prevalence rates of toxoplasmosis in pigs, cattle, goats, sheep, donkeys, horses, chickens, and ostriches slaughtered for human consumption were 77%, 48.3%, 42.9%, 44.8%, 8.65%, 47.2%, 69.8%, and 80%, respectively (Tables S3–S5).

We found studies including animals from the North (n = 4), Northeast (n = 28), South (n = 17), Southeast (n = 16), and Midwest (n = 2) regions. The highest prevalence values found in the Brazilian regions were: 50% in pigs in the North [36], 69.8% in chickens in the Northeast [57], 48.3% in cattle in the South [44] and 80% in ostriches in the Southeast [60].

**Figure 3:**
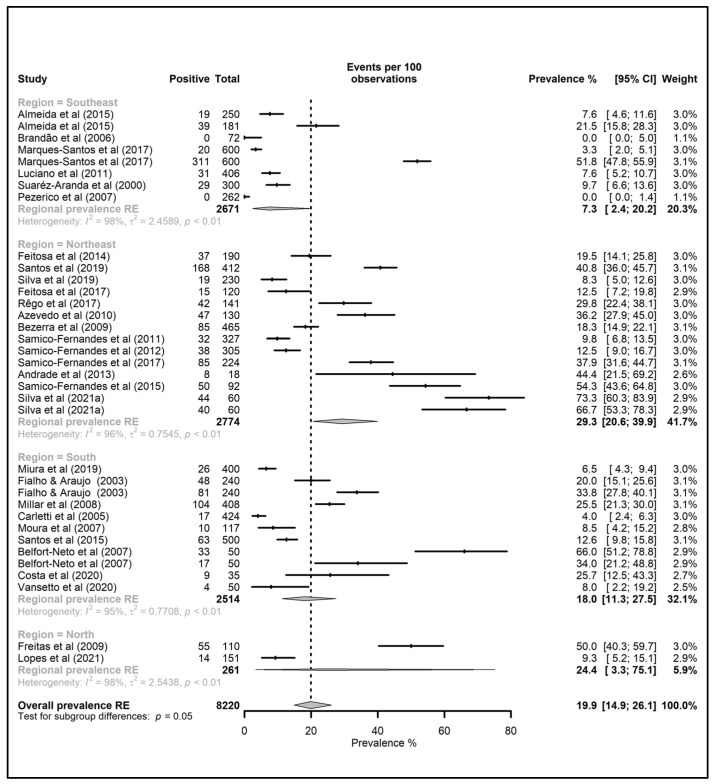
Forest tree of studies reporting toxoplasmosis prevalence in pigs in Brazil, grouped by administrative region (North, Northeast, Southeast and South). CI: confidence interval; RE: random effect.

**Figure 4:**
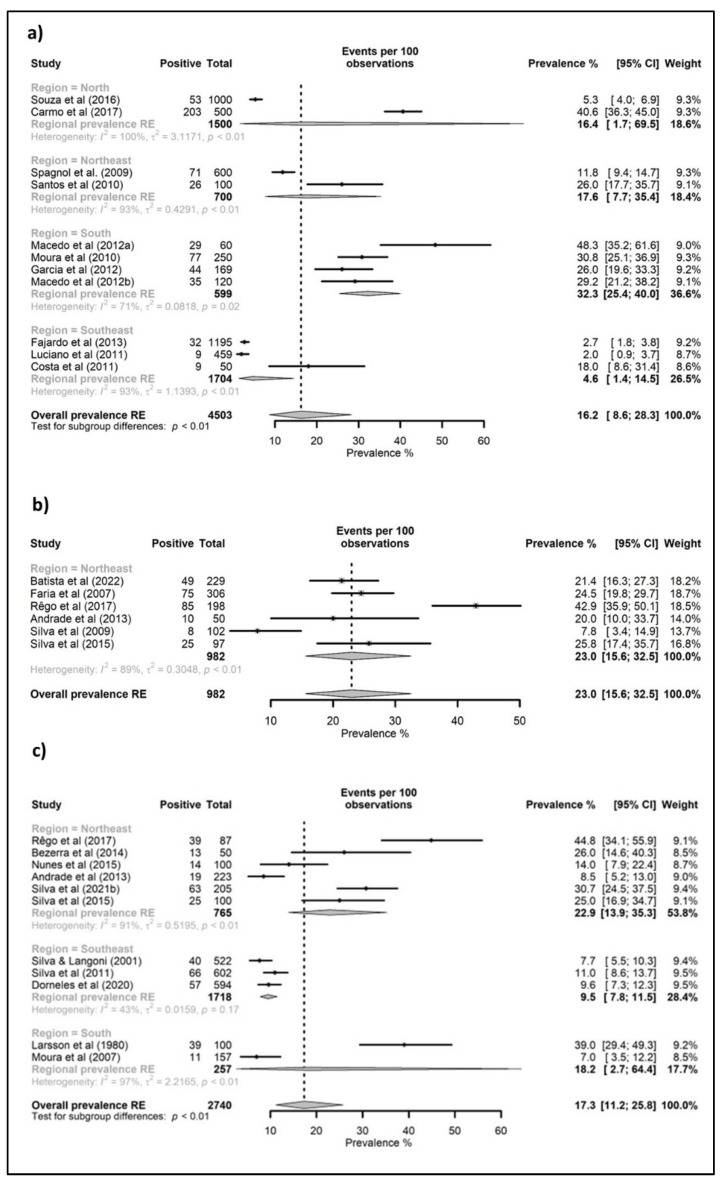
Forest tree of studies reporting toxoplasmosis prevalence in ruminants slaughtered for human consumption in Brazil, grouped by administrative region. (**a**) Cattle, (**b**) goats, and (**c**) sheep. C.I: confidence interval; RE: random effect.

**Figure 5:**
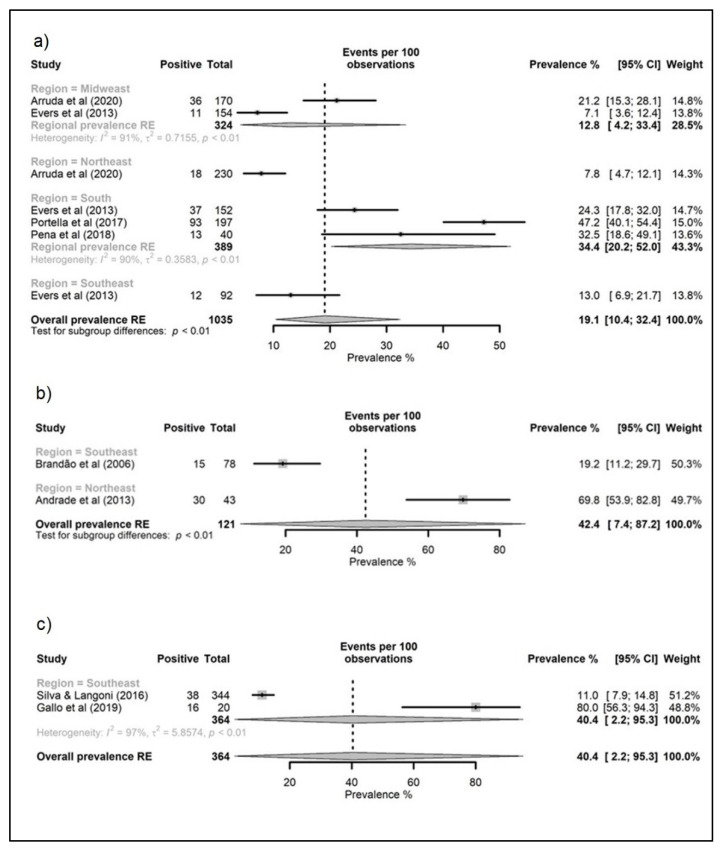
Forest tree of studies reporting toxoplasmosis prevalence in equids and avian slaughtered for human consumption in Brazil, grouped by administrative region. (**a**) Equids, (**b**) chickens, and (**c**) ostriches. CI: confidence interval; RE: random effect.

The meta-analyses’ results regarding the overall prevalence estimates of pigs, cattle, goats, sheep, equids, poultry, and ostriches slaughtered for human consumption in Brazil and its regions are shown in Figure 3, Figure 4 and Figure 5. The highest overall prevalence values were seen for poultry and ostriches (42.4 and 40.4%, respectively), but there were only two studies for each species. Additionally, the overall prevalence for goats, swine, equids, sheep, and bovine was 23.0, 19.9, 19.1, 17.3, and 16.2%, respectively. Regarding the data for each species in the Brazilian regions, high prevalence values were estimated for bovines and equids in the South (32.3 and 34.4%, respectively); swine, goats, sheep, and poultry in the Northeast (29.3, 23.0, 22.9, and 69.8%, respectively); and ostriches in the Southeast (40.4%). All studies with goats were performed in the Northeast region, while those evaluating ostriches were conducted in the Southeast.

Although seropositive animals may have infective tissue cysts, other methodologies were used in other studies to assess that. We found 22 manuscripts (Table S6) that used tissue, mainly from seropositive animals (except studies 9, 10, 12, 17, 18, 20, and 21), to perform bioassays or PCR to verify the presence of infective cysts or parasite DNA. The highest viability proportions in bioassays were seen for sheep (34/40 = 85%) [26] and swine (12/15 = 80%) [24]. The viability had a large range from 0% to 85% (Table S6). Two studies found seropositive animals (sheep) that later were all negative in the bioassay [25,57].

The risk factors for toxoplasmosis in animals slaughtered in Brazilian abattoirs were described in 16 manuscripts, covering pigs (n = 7), horses/donkeys (n = 1), goats (n = 1), sheep (n = 1), cattle (n = 5) and ostriches (n = 1) (Table 1). There was no risk factor described for poultry slaughtered for human consumption in Brazil. The risk factors reported in more than one manuscript were animals raised in extensive systems, the presence of cats, older animals, female animals, and the area where animals originated (Table 1).

## 4. Discussion

*Toxoplasma gondii* is considered one of the most successfully adapted parasites, infecting many species, including mammals and avians [69], resulting in economic losses for farmers and disease burden in humans [70]. Clinical toxoplasmosis is relatively infrequent, but around 30% of the human population is considered infected with the parasite. Despite the worldwide distribution, most outbreaks of this disease occur in Brazil, and many are associated with contaminated meat consumption. In addition to the importance of meat as a vehicle for this parasite, there were many large outbreaks in Brazil related to the consumption of water or food (mainly vegetables) contaminated with oocysts shed by felids [5].

Humans who eat raw or undercooked meat are exposed to the risk of ingesting *T. gondii* tissue cysts when animals are infected. Eating raw or undercooked meat led to 1.2–1.3 times the risk and 1.7–3.0 times the odds of toxoplasmosis, respectively, regardless of the species they consume [71]. The European Food Safety Authority (EFSA) estimated that meat accounts for about 60% of toxoplasmosis transmission and mainly occurs through consuming pork, beef, and small ruminant meat [72]. Despite the importance of this transmission route, meat inspection conducted at abattoirs fails to detect infected animals, as production animals will rarely present macroscopic lesions or clinical signs at abattoirs [73]. There are no reports of the presence of clinical signs in animals in any of the manuscripts included in this systematic review. However, the high number of seropositive animals and their infectivity reinforces the importance of meat in toxoplasmosis transmission.

According to the meta-analyses of Belluco and colleagues [74], the *T. gondii* worldwide prevalence for cattle, pigs, and sheep was estimated at 2.6%, 12.3%, and 14.7%, respectively. In the meta-analyses presented in this manuscript, the overall prevalence estimates for goats, pigs, equids, sheep, and cattle were generally higher, with 23.0, 19.9, 19.1, 17.3, and 16.2%, respectively. Although the whole of South America is considered a high-prevalence area [75], the occurrence of toxoplasmosis varies across the different geographic regions. For instance, this study on the Brazilian regions (Figure 3, Figure 4 and Figure 5) brings to light the higher prevalence values for bovines and equids in the South, and for swine, goats, sheep, and poultry in the Northeast.

A factor that could contribute to our higher results was the selection criteria used in this study. We selected only studies performed on animals slaughtered for human consumption in abattoirs. Commonly, these animals are older than a mixed population sampled on farms. Older animals have a higher risk of being infected with *T. gondi* [47]. Additionally, the heterogenicity of the collected data may have affected the meta-analyses results [74].

Pork is considered a major source of *T. gondii* infection [2], which may explain the high number of studies focused on pigs in Brazil (30 studies). According to the meta-analyses presented in this study, an overall prevalence of 19.9% (C.I. 60.3–83.9%) was established, with values ranging from 0 to 73.3%. Numerous studies collected tissues from seropositive pigs and evaluated their infectivity using bioassay with a proportion of positive animals ranging from 2 to 69.2% [21,61] (Table S6). Even though high hygiene standards have been adopted in swine farms in Brazil, outdoor raising still occurs in the country. These animals are more likely to acquire toxoplasmosis through ingesting contaminated food and water, and through access to small animals such as rodents [19]. Thus, prophylactic strategies such as avoiding rodents in piggeries [16,20], proper disposal of dead animals [16,22], indoor husbandry [22,31], avoidance of feeding with leftovers [19] or contaminated water [16,20,22], all commonly indicated risk factors (Table 1), must be more implemented in Brazilian swine farms.

The meat from small ruminants (sheep and goats) and equids is also important in the foodborne transmission of *T. gondii* [2]. The overall prevalence in goats and sheep was 23.0 and 17.3%, respectively. According to Belluco and colleagues [74], *T. gondii*’s global prevalence in sheep is 14.7%, agreeing with this study. The selling of infected lamb may expose consumers to infection risk. This was also shown by the study of Plaza and colleagues [76], in which 6 of 87 (6.9%) retail meat samples in Scotland tested positive for *T. gondii* DNA using PCR. The infective potential of lamb meat in Brazil was assessed using bioassay, PCR and immunohistochemistry (Table S6), with the positive proportion ranging from 0 [25] to 85% [26], evidencing this potential for foodborne transmission. Here also, avoiding extensive breeding systems where animals have outdoor access can contribute to the reduction in *T. gondii* infections in small ruminants [63].

Meat consumption from equids and ostriches is very rare in Brazil compared with other meats (swine, bovine, and poultry), and probably results in a low risk in the country. Nevertheless, Pena and colleagues [52] isolated *T. gondii* from horse meat in a Brazilian abattoir destined for export to Europe, emphasizing the need for controlling this parasite. The species (horses have a higher risk than donkeys), animal origin (geographic area), the purpose of rearing (meat production), the source of drinking water (riverside), and contact with cats have been identified as risk factors and could be suggested as a focus of sanitary programs [11].

Commonly, the prevalence of *T. gondii* in beef is low, and its risks have been debated due to the low number of positive samples in bioassays [2]. Still, beef may be an important source of infection, as it is frequently consumed undercooked in Brazilian areas, and while the overall prevalence in cattle was 16.2% (8.6–28.3%), the South region presented a higher estimate of 32.3% (C.I. 25.4–40%). In Brazil, Macedo and colleagues [44] reported 26.6% (16/60) positivity using blood or fetuses in a bioassay, and Santos and colleagues [62] reported a proportion of 7.7% (2/26) positive brain or heart samples using PCR. Risk factors identified for cattle included animals raised on a feeder/stocker/backgrounder system, the presence and number of resident/stray cats, the presence of cats walking freely in farms, rat control by using cats, feed storage, and age (older) [29,47,50].

Only two studies included chickens, which resulted in an overall prevalence estimate of 42.4% (Figure 5). Additionally, infective samples were detected in a bioassay using contaminated poultry [57]. However, poultry is not considered high-risk meat since it is consumed completely cooked and frequently sold frozen, reducing the risk of foodborne transmission [2].

The aforementioned factors for different animal species can be implemented on farms to avoid the transmission of toxoplasmosis via meat. Establishing monitoring programs focused on reducing environmental contamination is a pivotal strategy [73]. Generally, the recommended measures include avoiding the access of rodents near omnivores or cats for any farm animal, biosecurity practices, and a restrictive policy of restocking animals [70]. Further down the food chain, decontamination procedures, such as freezing for several days or cooking, may contribute to minimizing the infection risk for people. Educational programs targeting different stakeholder groups can support this [77]. The Centers for Disease Control and Prevention (CDC) recommend cooking at temperatures of at least 145 °F (63 °C), 160 °F (71 °C), and 165 °F (74 °C) for whole cuts of meat, ground meat, and poultry, respectively. Cysts of *T. gondii* can be inactivated during salt curing of meat products, but this depends on the maturation time, temperature, and salt concentration [1].

## 5. Conclusions

The high prevalence of *T. gondii* in Brazilian livestock varies within species and regions, but it is widespread in the country. There is a need for controlling this food-borne zoonosis, and the summarized data allow the establishment of high-priority areas and/or species, such as bovines and equids in the South and swine, goats, sheep, and poultry in the Northeast. More studies are required to fully comprehend its epidemiology, mainly in the Midwest region, and the effectiveness of preventive strategies, including animals, humans, and ecosystems, to control this parasite at different levels of the Brazilian meat chain.

## Figures and Tables

**Table 1:** Risk factors for toxoplasmosis infection in animals slaughtered for human consumption in Brazil.

Number	Species	Risk Factor	Reference	Reference Number
1	Bovine	Animals raised on a feeder/stocker/backgrounder system	Souza et al. (2016)	[29]
2	Bovine	The presence and number of resident cats, presence and number of stray cats, presence of cats walking freely, rat control by using cats and feed storage	Fajardo et al. (2013)	[50]
3	Bovine	Age (more than two years) and not pure breed	Moura et al. (2010)	[66]
4	Bovine	Age (older)	Garcia et al. (2012)	[47]
5	Bovine	Pregnant and breed (Jersey compared to Holstein)	Macedo et al. (2012b)	[65]
6	Goat	Animal origin (municipalities)	Batista et al. (2022)	[9]
7	Ostriches	Water tank and presence of non-ostrich feces in paddocks	Silva & Langoni (2016)	[27]
8	Sheep	Females, extensive breeding system, and purebred animals	Silva et al. (2021b)	[63]
9	Swine	Type of animal Inspection Service in slaughterhouse (Federal), the state that originated the animals, the presence of rats in the piggery, the origin of the used water, hygienic and sanitary condition of piggery, routing of waste and disposal of the dead animals	Almeida et al. (2015)	[16]
10	Swine	Extensive husbandry and feeding with leftovers	Feitosa et al. (2014)	[19]
11	Swine	Lineage, animal origin, size of the farm (larger ones), collective raising with others species (bovine), presence of rodents and type of water offered	Marques-Santos et al. (2017)	[20]
12	Swine	Age (higher than six months)	Silva et al. (2019)	[23]
13	Swine	Animal sex (female), raising system (confined) and origin of the animals (municipality)	Bezerra et al. (2009)	[31]
14	Swine	Female gender, semi-confined rearing system, use of well water, dewormed animals, presence of cats, goats, sheep, mice and vultures on the farm and carcasses left on the ground	Santos et al. (2019)	[22]
15	Swine	Age (older)	Samico-Fernandes et al. (2017)	[54]
16	Horse	Specie (horse higher than donkey), animal origin (state), purpose of rearing (meat production) and source of water for animal consumption (riverside) and contact with cats	Arruda et al. (2020)	[11]
	Donkey			

## Data Availability

Full data are available in the references list and Supplementary Files, mainly Supplementary File S1.

## Supplementary Materials

The following supporting information can be downloaded at: https://www.mdpi.com/article/10.3390/ani12223102/s1, Table S1: PRISMA Checklist; Table S2: Raw data of systematic review; Table S3. The set of studies including pigs slaughtered for human consumption in Brazil; Table S4. The set of studies including ruminants slaughtered for human consumption in Brazil; Table S5. The set of studies including equids and avian slaughtered for human consumption in Brazil; Table S6. The set of studies that evaluated the infectious potential of animals slaughtered in Brazil for human consumption.
